# Biomechanical properties of human thoracic spine disc segments

**DOI:** 10.4103/0974-8237.65477

**Published:** 2010

**Authors:** Brian D. Stemper, Derek Board, Narayan Yoganandan, Christopher E. Wolfla

**Affiliations:** Department of Neurosurgery, Medical College of Wisconsin, Milwaukee, WI, USA

**Keywords:** Biomechanics, modulus, segmental properties, spine, stiffness

## Abstract

**Background:**

The objective was to determine the age-dependent compressive and tensile properties of female and male thoracic spine segments using postmortem human subjects (PMHS).

**Materials and Methods:**

Forty-eight thoracic disc segments at T4-5, T6-7, T8-9, and T10-11 levels from 12 PMHS T3-T11 spinal columns were divided into groups A and B based on specimen age and loaded in compression and tension. Stiffness and elastic modulus were computed. Stiffness was defined as the slope in the linear region of the force—displacement response. Elastic modulus was defined as the slope of the stress strain curve. Analysis of Variance (ANOVA) was used to determine significant differences (*P*<0.05) in the disc cross-sectional area, stiffness, and elastic modulus based on gender, spinal level, and group.

**Results:**

Specimen ages in group A (28 ± 8 years) were significantly lower than in group B (70 ± 7 years). Male discs had significantly greater area (7.2 ± 2.0 sq cm) than female discs (5.9 ± 1.8 sq cm). Tensile and compressive stiffness values were significantly different between the two age groups, but not between gender and level. Specimens in group A had greater tensile (486 ± 108 N/mm) and compressive (3300 ± 642 N/mm) stiffness values compared to group B specimens (tension: 397 ± 124 N/mm, compression: 2527 ± 734 N/mm). Tensile and compressive elastic modulus values depended upon age group and gender, but not on level. Group A specimens had significantly greater tensile and compressive moduli (2.9 ± 0.8 MPa, 19.5 ± 4.1 MPa) than group B specimens (1.7 ± 0.6 MPa, 10.6 ± 3.4 MPa). Female specimens showed significantly greater tensile and compressive moduli (2.6 ± 1.0 MPa, 16.6 ± 6.4 MPa) than male specimens (2.0 ± 0.7 MPa, 13.7 ± 5.0 MPa).

**Discussion:**

Using the two groups to represent "young" and "old" specimens, this study showed that the mechanical response decreases in older specimens, and the decrease is greater in compressive than distractive properties. While the decrease is expected, the relative change between the two modes of loading has not been reported. Another conclusion from the study is that the mechanical properties depend on gender, although not as decisive due to sample size.

## INTRODUCTION

Under normal circumstances, the function of the human vertebral column is to protect neural structures by undergoing deformations while preserving normal interrelationships between its elements. For example, cervical spine segments deform due to head movements, and the dorsal spine deforms due to trunk motion. Deformations induced in the intervertebral segments are specific to loads, i.e. disc compression occurs due to compressive loads transferred from adjacent vertebrae. The presence of the head mass induces physiologic compression to cervical discs, albeit eccentric to the center of the cervical column. Likewise, axial loads on the dorsal spine (e.g., trunk mass inducing forward bending) bring about compressive forces in the anterior and tensile forces in the posterior columns.[1,2] Characterization of load-deformation responses as a function of vertebral column region assists in an improved understanding of load sharing among its various segments.

Many studies have been conducted to determine the load carrying capacity of spinal segments under externally applied forces. For example, the axial compressive force-deformation properties of human cadaver cervical and lumbar spines have been determined.[3‐8] Because the application of bending moments to a spinal segment results in local compression and tension in the same intervertebral joint, tensile properties are also of interest. Flexion moment induces tensile forces and stresses/strains to components dorsal to the neutral axis and compressive forces and stresses/strains to components ventral to this axis. The load carrying capacity of the human cervical and lumbar spines under axial tension has been investigated.[6‐13] Contributions of the posterior elements to cervical spine compressive and tensile stiffness values have also been determined.[14,15] Studies using the thoracic and lumbar regions of the spinal column have been conducted to determine its stability under compressive loads.[16,17] Although not exhaustive, these studies have determined the responses of the three mobile regions of the vertebral column under different loading modes.

Demographic factors, such as age, influence the load carrying capacity of biological materials. For example, the compressive strength of lumbar vertebral bodies depends on its bone mineral content, and decreases with increasing age.[18] The effect of gender on the bone mineral content and load carrying capacity of vertebral bodies has been reported.[19‐23] Similar studies examining the interrelationship between the compressive and tensile stiffness and the elasticity properties of vertebral body-disc-vertebral body units (disc segments) and factors such as gender and spinal level have not been conducted. This is important because the disc is more deformable than the vertebral body, and, as described, the spinal column sustains external forces through internal deformations of its components. The objective of this study was therefore to determine the effect of gender and spinal level on the properties of age-dependent thoracic spine segments using postmortem human subjects (PMHS).

## MATERIALS AND METHODS

Twelve PMHS T3-T11 spines (six males and six females) were divided into two groups: group A consisted of specimens with age less than 40 years, and group B consisted of specimens with age more than 60 years. Spines were sectioned into 48 disc segments at T4-5, T6-7, T8-9, and T10-11 levels. Posterior elements were removed dorsal to the pedicles, including facet joints, laminae, spinous processes, and associated ligaments. Specimens were aligned such that the mid-disc plane was horizontal and fixed at the superior and inferior ends using methylmethacrylate. The disc height was measured at the anterior and right/left lateral aspects using a digital micrometer.

The superior fixation was attached to the piston of a custom-designed electro-hydraulic testing device (MTS Systems Corp., Eden Prairie, MN, USA), and the inferior fixation was attached to the loading frame through a load cell mounted on an *x-y* cross table. Specimens were preconditioned for five cycles and loaded in tension or compression to 25% of the mean unstressed disc height. Each specimen was subjected to three loading cycles of compression and three loading cycles of tension in randomized order. Following testing, specimens were sectioned at the mid-disc height, photographs were obtained, and intervertebral disc cross-sectional areas were measured (Digital image analysis, ACD Systems of America Inc., Miami, FL).

**Figure 1:**
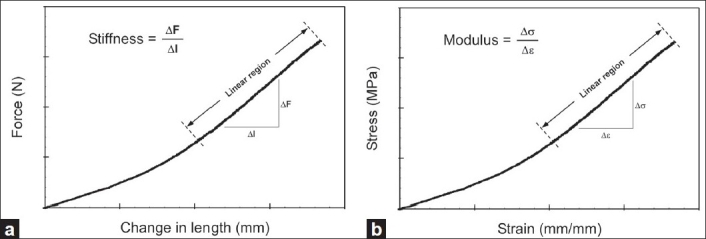
(a) Force-displacement curve used to compute stiffness (b) Stress-strain curve used to compute elastic modulus

Compressive and tensile stiffness values were computed as the slope in the linear region of the force-displacement curve [Figure 1]. Stress was defined as the axial force divided by disc cross-sectional area. Strain was defined as the displacement divided by unstressed disc height. Elastic modulus was defined as the slope in the linear region of the stress-strain curve. Analysis of Variance (ANOVA) determined significant (*P*<0.05) differences in stiffness and elastic modulus based on gender, spinal level, and age group. Mean stiffness and elastic modulus across all three repeated trials were used for statistical comparisons.

## RESULTS

The experimental design was such that specimen ages in group A (28 ± 8 years) and B (70 ± 7 years) were significantly different [Table 1]. Disc areas in male specimens depended upon gender and level; for the entire ensemble, males had greater area (7.2 ± 2.0 sq cm) than female (5.9 ± 1.8 sq cm) specimens. Disc heights did not show any such tendency (male specimens: 4.3 ± 0.8 cm and female specimens: 4.2 ± 0.6 cm).

**Table 1:** Summary of demographic and geometry data (mean and standard deviation)

Group	Age (Years)	Height (cm)	Body mass (kg)	BMD (gm/cc)	Disc height (cm)	Disc area (sq cm)
A	Male	30 ± 6	180 ± 3	84 ± 26	150 ± 38	4.7 ± 0.9	7.2 ± 2.1
	Female	27 ± 10	155 ± 18	48 ± 16	171 ± 22	4.4 ± 0.4	5.6 ± 1.9
	All	28 ± 8	170 ± 17	70 ± 28	161 ± 33	4.5 ± 0.6	6.4 ± 2.1
B	Male	69 ± 9	179 ± 13	75 ± 12	108 ± 34	3.8 ± 0.4	7.2 ± 2.0
	Female	70 ± 7	164 ± 12	79 ± 50	107 ± 25	4.1 ± 0.7	6.3 ± 1.7
	All	70 ± 7	172 ± 14	77 ± 33	107 ± 29	4.0 ± 0.5	6.7 ± 1.9
A + B	All	49 ± 23	171 ± 15	74 ± 29	134 ± 41	4.2 ± 0.6	6.6 ± 2.0
	All males	50 ± 23	180 ± 8	80 ± 19	129 ± 41	4.3 ± 0.8	7.2 ± 2.0
	All females	48 ± 25	160 ± 13	66 ± 40	139 ± 40	4.2 ± 0.6	5.9 ± 1.8

**Figure 2:**
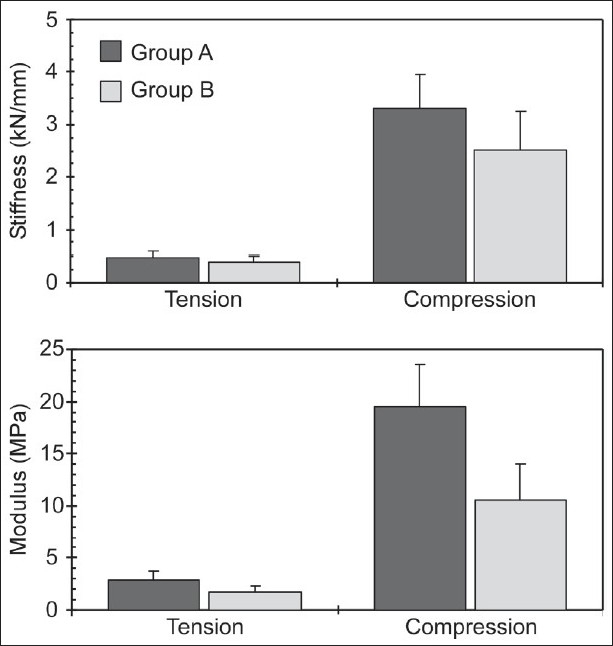
Stiffness and modulus separated by age group

Tensile and compressive stiffness values were significantly different between the two age groups, but not significantly dependent upon gender and level [Table 2, Figure 2]. There were no significant statistical interactions between any combination of gender, group, and level for both parameters. Group A specimens demonstrated greater tensile (486 ± 108 N/mm) and compressive stiffness (3300 ± 642 N/mm) compared to group B (tension: 397 ± 124 N/mm, compression: 2527 ± 734 N/mm) specimens. Greater compressive stiffness in males than females in each group did not reach statistical significance.

**Table 2:** Summary of biomechanical properties (mean and standard deviation)

Group		Tensile stiffness (N/mm)	Compressive stiffness (N/mm)	Tension modulus (MPa)	Compressive modulus (MPa)
A	Male	478 ± 141	3395 ± 609	2.5 ± 0.7	17.7 ± 3.4
	Female	494 ± 66	3205 ± 686	3.3 ± 0.6	21.3 ± 4.1
	All	486 ± 108	3300 ± 642	2.9 ± 0.8	19.5 ± 4.1
B	Male	406 ± 85	2737 ± 633	1.4 ± 0.3	9.7 ± 2.6
	Female	387 ± 161	2297 ± 797	1.9 ± 0.7	11.5 ± 4.1
	All	397 ± 124	2527 ± 734	1.7 ± 0.6	10.6 ± 3.4
A + B	All	442 ± 123	2922 ± 785	2.3 ± 0.9	15.1 ± 5.9
	All males	442 ± 120	3066 ± 694	2.0 ± 0.7	13.7 ± 5.0
	All females	443 ± 130	2771 ± 860	2.6 ± 1.0	16.6 ± 6.4

**Figure 3:**
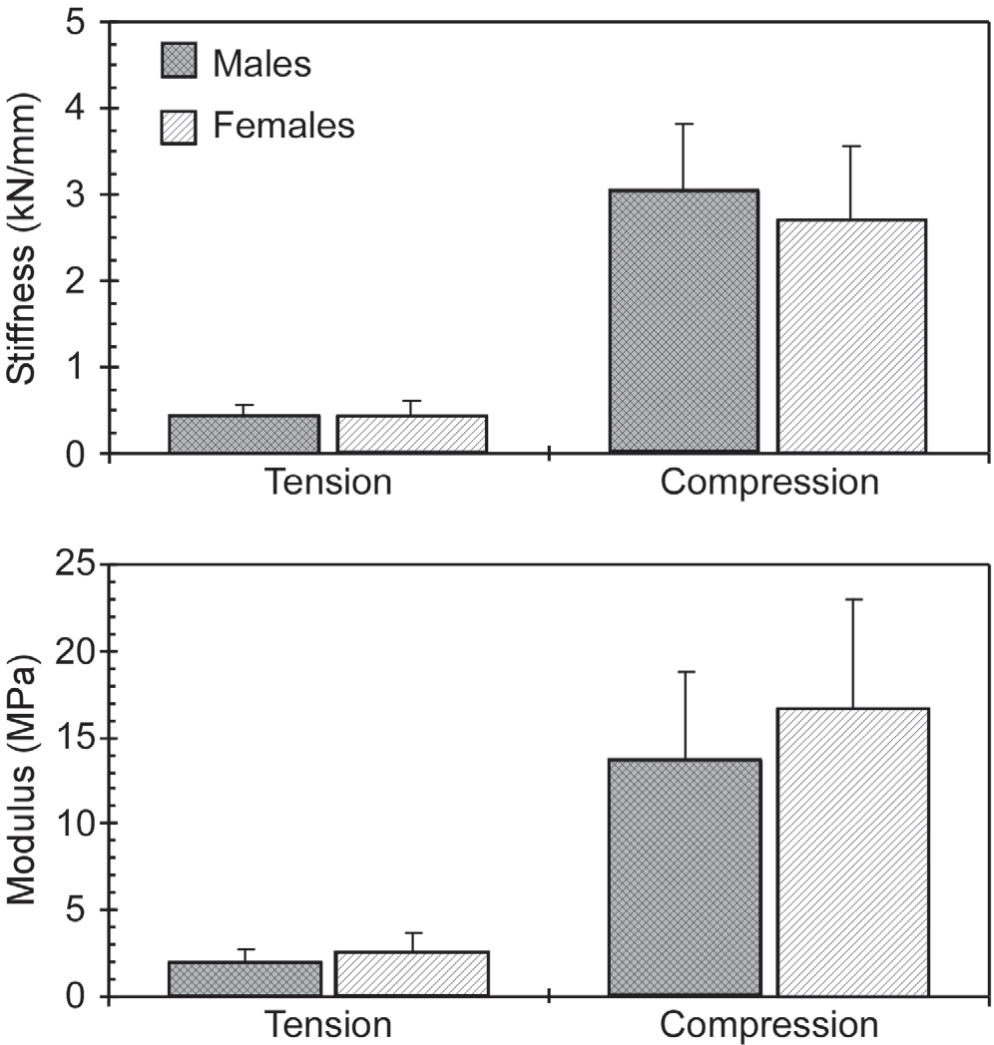
Stiffness and modulus separated by gender

Tensile and compressive elastic modulus values depended upon age group and gender, but not upon level [Table 2, Figure 3]. No significant statistical interactions were found between any combination of gender, age, and level for both parameters. Group A specimens demonstrated significantly greater tension modulus (2.9 ± 0.8 MPa) than group B specimens (1.7 ± 0.6 MPa). In contrast to stiffness, female specimens demonstrated significantly greater tension modulus (2.6 ± 1.0 MPa) than male (2.0 ± 0.7 MPa) specimens. Likewise, group A specimens demonstrated significantly greater compression modulus (19.5 ± 4.1 MPa) than group B specimens (10.6 ± 3.4 MPa), and female specimens demonstrated significantly greater compression modulus (16.6 ± 6.4 MPa) than male specimens (13.7 ± 5.0 MPa). Figure 3 and Table 2 show variations in stiffness and modulus as a function of gender for the entire ensemble. While the modulus was found to be significantly different, stiffness did not show this trend.

## DISCUSSION

The experimental design was such that the two groups represented statistically different ages, reflecting the selection bias. The two selected age groups reasonably represented mature adult "young" and "old" populations. This observation is based on the fact that the compressive and tensile properties of adult human intervertebral discs decrease with age from the younger (20-39 years of age) populations.[24] The mean ages of specimens in the present study for groups A and B were 28 and 70 years [Table 1]. It is appropriate to expect larger differences if group B specimens were older, e.g. in the eighth decade. However, such analysis would require a large number of specimens and is beyond the scope of the present study. Further, the applicability of such elderly population data may be limited because of their decreased activity. A much larger sample size would be required to determine mechanical properties as a function of age due to the continuous development, maturation, and degeneration of spinal components. Such a protocol could delineate the specific age group at which properties begin to show (significant) age dependence.

With regard to tensile and compressive stiffness data, tensile magnitudes from the present study were found to be lower than previous data.[8,16,25] However, compressive stiffness was similar to literature values.[8,16,25] The discrepancy between them may be due to the relative contribution of spinal components in specific loading modes. During compressive loading of the cervical spine, approximately 75% of the applied load is transmitted through the disc, with the remaining supported by facet joints.[14,15] The ratio is likely higher in the thoracic spine due to the vertical orientation of facet joints. However, posterior elements and ligaments play a larger role in supporting tensile loads. The removal of facet joints and ligamentum flavum to obtain disc segments used in the present study may have contributed to the considerable decrease in tensile stiffness when compared to motion segment outputs [Table 3].

**Table 3:** Comparison with literature

Study	Tensile stiffness (N/mm)	Compressive stiffness (N/mm)
Markolf [8]	700 - 1580	1230 - 3320
Panjabi *et al* [25]	758	1250
Yoganandan *et al* [16]	-	3240 ± 450
Yoganandan *et al* [16]	-	2030 ± 330

Previous investigations using thoracic motion segments did not identify significant gender differences presumably due to inclusion of a smaller number of female specimens,[8] a sample size of one at each level,[25] or undisclosed gender information.[16] In contrast, the present experimental design controlled for gender, thus making this variable a part of the statistical analyses. While the difference was statistical, additional studies are needed to understand its implications, especially in surgical treatment. While indications for surgical treatment are not generally gender-specific, gender has been implicated as a risk factor in certain postoperative complications.[26] It remains to be seen whether the biomechanical differences described in the present study represent an intrinsic risk factor separate from those that have been previously defined.

Greater disc cross-sectional areas in male than female specimens, and with no such differences in the height parameter, indicate that the intervertebral disc volume is greater in males. These geometrical properties appear to have bearing on the load carrying capacity of the disc segment. Additionally, these differences are more pronounced in compression than tension, likely because of the lesser or nonexistent role of the anterior ligaments in the compressive mode. For a given compressive or tensile displacement, the present study shows that male specimens sustain/resist a greater force than the female counterpart, resulting in greater stiffness. In contrast, the lesser disc cross-sectional area in the female results in greater stress, tensile or compressive, than male specimens, though the resistive force is lower in females. Acknowledging that strain levels were the same for both male and female specimens, increased elastic modulus in females stems from greater stresses. In other words, the ratio of the increase in force in males with respect to females contributing to the stress variable has a lesser effect than the increased ratio of disc area in males with respect to females. This may imply an accentuated role of the disc area in females, more pronounced in compression than tension. Thus, it appears that the axial plane geometry may be a factor for the gender effect in elastic modulus for females.

The present result, i.e. compressive stiffness greater than the tensile stiffness is different from the cervical spine, perhaps reflecting the anatomical differences between the two regions.[14] Under axial tension, the disc and two longitudinal ligaments contribute to the load carrying capacity. Using earlier studies as a basis, the overall stiffness of the two ligaments can be estimated to be approximately 50 N/mm.[27] Assuming linearity principles and results from this study [Table 1], the intact thoracic disc offers approximately 400 N/mm resistance to this disc segment. Any compromise of the integrity of the annulus or nucleus components may decrease this load carrying capacity. These types of analyses may have clinical implications in procedures such as discectomy or nucelectomy, partial or full. A more quantitative analysis could be done by extending these experiments to include specimens under intact intervertebral joint, disc joint (as done in the present study), and varying levels of nucelectomy and discectomy conditions.

## CONCLUSION

Results from the present study showed demographic effects on the biomechanical properties of thoracic spine segments. Using the two groups to represent "young" and "old" specimens, this research showed that the mechanical response decreases in older specimens, and further, the decrease is greater in compressive than distractive properties [Figures 2 and 3]. While a decrease is expected, the relative change between the two modes of loading has not been reported. Another conclusion from the present study is that the mechanical properties depend on gender, although not as decisive due to sample size.
